# The Prevalence of Coronary Artery Disease and Its Prognostic Impact on the Management of Patients With Acute Decompensated Heart Failure: A Prospective Cohort Study With a Short-Term Follow-Up

**DOI:** 10.7759/cureus.71717

**Published:** 2024-10-17

**Authors:** Raed A Alnutaifi, Fayez Elshaer, Ghaliah S Alnefaie, Talal S Abozaid, Ghada Alharbi, Manal Altwaim, Omar Alharbi, Mohammed Alqhtani, Naif A Alshehri

**Affiliations:** 1 College of Medicine, King Saud University, Riyadh, SAU; 2 Department of Cardiac Sciences, College of Medicine, King Khalid University Hospital, King Fahad Cardiac Center, King Saud University, Riyadh, SAU; 3 Department of Cardiology, National Health Institute, Cairo, EGY; 4 College of Medicine, King Saud University for Health Sciences, Riyadh, SAU

**Keywords:** coronary artery disease, heart failure, hospitalizations, morbidity, mortality, outcome

## Abstract

Introduction

Heart failure (HF) poses a major global health challenge, with acute decompensated heart failure (ADHF) representing a critical phase that requires immediate medical intervention. Coronary artery disease (CAD) plays a significant role in many HF cases, contributing to disease progression through myocardial ischemia and impaired ventricular function. While the connection between CAD and HF is well-established, its specific effect on short-term outcomes in patients with ADHF is less understood, especially in regions like the Middle East. This study aims to evaluate the influence of CAD on short-term outcomes in patients presenting with ADHF and identify key differences in demographics, clinical parameters, and outcomes, including intensive care unit (ICU) admissions and medications, between patients with and without CAD, addressing gaps in current understanding and offering insights to improve clinical management.

Methods

This prospective cohort study was conducted at King Saud University Medical City (KSUMC) in Riyadh, Kingdom of Saudi Arabia (KSA), from April 2023 to April 2024. We included a total of 144 known heart failure patients presenting with acute heart failure (AHF) to the emergency department (ED). Secondary data was collected from the KSUMC medical records database to track patient outcomes after six months. Significant coronary angiography lesions, defined as 70% stenosis or greater, or a history of myocardial ischemia were necessary as evidence of CAD to meet the inclusion criteria. Statistical analyses were conducted using Chi-squared tests for categorical variables and t-tests for continuous variables. All analyses were performed using RStudio version 4.3.1 (Posit Software, Boston, MA), with a significance threshold set at p < 0.05.

Results

The study included 144 known heart failure patients presenting with acute decompensation, with 83 (57.6%) patients having CAD. CAD patients were younger (median age: 66 versus 67 years, p = 0.026) and predominantly male (75.9% versus 59%, p = 0.031). Diabetes mellitus was significantly more prevalent among patients with CAD (74.7% versus 49.2%, p = 0.002). Ejection fraction (EF) was notably lower in the CAD group with a greater proportion having an ejection fraction of 40% or less (89.2% versus 62.3%, p < 0.001). Additionally, CAD patients had more frequent ICU admissions (37.3% versus 13.1%, p = 0.001) and were more likely to present with chest pain (38.6% versus 21.3%, p = 0.027), while weight loss and lower extremity edema were more common in non-CAD patients (p < 0.05). Despite these worse clinical signs, CAD patients did not have significantly higher mortality at 180 or 360 days.

Conclusion

Although there was no statistically significant difference in mortality, CAD patients exhibited more severe disease indicators, such as lower ejection fractions and increased ICU hospitalizations. These findings underscore the importance of early detection and tailored treatment strategies for CAD in ADHF patients. Clinicians should prioritize aggressive management of CAD to prevent disease progression and reduce the need for ICU admissions. Future studies should focus on long-term outcomes and explore the impact of specific interventions, such as early revascularization or optimized heart failure therapies, to better understand how CAD influences ADHF prognosis.

## Introduction

Heart failure (HF) is a serious global health burden, affecting an estimated 26 million people worldwide, and is becoming more common [1]. This burden is particularly pronounced in our Middle East region with an estimated prevalence of roughly 3.75 million people [2]. Also, specifically in Saudi Arabia, the local prevalence of HF has been rising over the years, and according to one study done in 2023, it reached up to 1.2% of the whole population [3].

Acute decompensated heart failure (ADHF) is a very important phase marked by a rapid worsening of symptoms in people with chronic HF, frequently requiring immediate urgent care [4]. Coronary artery disease (CAD), the leading cause of heart failure, is a condition in which the coronary arteries narrow or become blocked due to atherosclerotic plaque buildup, resulting in myocardial ischemia and infarction. The resulting ischemic episodes cause myocardial damage and contribute to left ventricular dysfunction, eventually leading to heart failure progression [4]. CAD is a critical therapeutic target for reducing the morbidity and mortality associated with HF as it can be the main etiological factor in 50% of patients [5]. CAD is an important contributor to HF exacerbations as repeated ischemic episodes can lead to adverse cardiac remodeling, increased ventricular stiffness, and eventually, the development of systolic or diastolic dysfunction [4].

The general management of ADHF typically includes a combination of diuretics, vasodilators, and inotropes aimed at relieving congestion, improving hemodynamics, and enhancing cardiac output [6]. However, the existence of CAD can make treatment plans more challenging, requiring the use of further treatments such as revascularization procedures, anti-ischemic drugs, or more strong control of associated conditions such as diabetes and hypertension [6].

Several randomized controlled trials (RCTs) have evaluated the prognostic role of CAD in HF. For instance, a study by Purek et al. found that CAD was a strong and independent predictor of mortality in patients with acute heart failure (AHF) [7]. In their prospective cohort study of 217 patients with acute congestive HF, they observed a significantly lower cumulative survival rate at 720 days in patients with CAD (48.7%) compared to those without CAD (76.4%, p = 0.0004). CAD increased the risk of death by 257% (hazard ratio (HR): 2.57, 95% confidence interval (CI): 1.50-4.39, p = 0.001), even after adjustments for age, sex, and comorbid conditions. These findings highlight that despite aggressive management, the presence of CAD significantly worsens outcomes in ADHF patients. The study also noted that underutilization of coronary revascularization procedures might have contributed to poor outcomes in CAD patients, underscoring the need for better therapeutic strategies tailored to this subgroup [7].

Although some RCTs such as that of Purek et al. have established CAD as a prognostic factor in acute heart failure, its impact on short-term outcomes in ADHF has not been extensively explored, particularly in Middle Eastern populations [7]. Therefore, this study aimed to evaluate the impact of CAD on short outcomes in patients presenting with acute decompensated heart failure to the emergency department (ED). Our study aimed to assess how coronary artery disease (CAD) affects the short-term outcomes of patients with acute decompensated heart failure (ADHF) in the emergency department. Additionally, the study sought to identify key differences in demographics, clinical parameters, and outcomes, including intensive care unit (ICU) admissions and medications, between patients with and without CAD. Addressing this knowledge gap could help optimize treatment strategies and improve patient outcomes.

## Materials and methods

Study design and setting

This prospective cohort study was carried out at King Saud University Medical City (KSUMC) in Riyadh, Saudi Arabia, over a one-year period from April 2023 to April 2024. The study aimed to explore the relationship between coronary artery disease (CAD) and short-term outcomes in patients presenting with acute heart failure. Additionally, the study sought to identify key differences in demographics, clinical parameters, and outcomes, including ICU admissions and medications, between patients with and without CAD. The research was conducted in the emergency department (ED) of KSUMC, a tertiary care center that serves a diverse patient population from Riyadh and surrounding regions.

Furthermore, as a tertiary care center, KSUMC receives referrals from different healthcare facilities across the region, enhancing the diversity and representativeness of the study population. The high patient volume and availability of a multidisciplinary team of specialists provide a unique opportunity to evaluate the prognostic impact of CAD on ADHF outcomes in a real-world clinical setting. These factors make KSUMC an ideal environment for conducting research on complex cardiovascular diseases, ultimately strengthening the study's generalizability and applicability to similar clinical settings.

Patient population and data collection

A total of 144 adult known heart failure patients presenting to the emergency department with symptoms of acute heart failure were enrolled in the study. Patients were selected based on clinical features (chest pain, weight loss, lower extremity edema, and heart rate) of acute decompensated heart failure (ADHF). Data was retrospectively gathered from the electronic health record system, e-Sihi, which is an integrated database containing comprehensive and up-to-date patient information. e-Sihi has been designed to ensure data completeness and reliability, providing real-time access to patient electronic health records. Medication data were verified through electronic health records and prescription logs within the e-Sihi system to ensure accuracy and completeness. The analysis focused on key variables such as age, sex, comorbidities (including hypertension, diabetes, asthma, chronic kidney disease (CKD), stroke, peripheral vascular disease, chronic obstructive pulmonary disease (COPD), and pneumonia), clinical symptoms, and laboratory parameters (ejection fraction (EF), glomerular filtration rate (GFR), and troponin I levels). Outcomes such as ICU admissions, prescribed medications at discharge (angiotensin-converting enzyme (ACE) inhibitors or angiotensin receptor blockers (ARBs), diuretics, glyceryl trinitrate, aspirin, and anticoagulants), and mortality rates at 180 and 360 days post-discharge were also examined to assess differences between patients with and without CAD. The inclusion of data such as comorbidities (e.g., diabetes, hypertension, asthma, and chronic kidney disease) was based on their established association with adverse heart failure outcomes, as reported in the literature [8].

Inclusion and exclusion criteria

To be included in the study, patients were required to meet specific criteria that indicated the presence of coronary artery disease (CAD). The criteria included having at least one of the following: a significant coronary lesion defined as a 70% or greater diameter stenosis observed on coronary angiography, a documented history of myocardial infarction, previous percutaneous coronary intervention (PCI), prior coronary artery bypass grafting (CABG), or evidence of myocardial ischemia during exercise stress testing. These criteria were selected to ensure that all participants had a confirmed and clinically significant diagnosis of CAD, providing a consistent baseline for evaluating its impact on acute decompensated heart failure (ADHF) outcomes.

The exclusion criteria were carefully chosen to avoid confounding factors that could independently impact mortality and morbidity. Patients presenting with cardiogenic shock were excluded due to their significantly higher risk of mortality, which could skew the study's evaluation of CAD's prognostic impact [9], as well as patients with severe renal disease, defined according to the Kidney Disease: Improving Global Outcomes (KDIGO) guidelines as chronic kidney disease (CKD) stage 4 or higher (serum creatinine level of 250 mmol/L or estimated glomerular filtration rate (eGFR) of <30 mL/minute/1.73 m²) [10]. This stage of renal impairment was chosen as an exclusion criterion because it is associated with significantly increased risks of mortality and adverse cardiovascular outcomes, which could confound the relationship between coronary artery disease (CAD) and heart failure outcomes [11]. By excluding these conditions, we aimed to reduce bias and ensure that the observed outcomes are primarily attributable to CAD-related factors, thus enhancing the study's robustness and internal validity.

Data analysis and statistical methods

Statistical analysis was performed using RStudio software (R version 4.3.) (Posit Software, Boston, MA) [12]. Continuous variables were compared across demographic groups using the Wilcoxon rank sum test, as these data were not normally distributed, which was confirmed by the Shapiro-Wilk test. Categorical variables were analyzed using Pearson's Chi-squared test or Fisher's exact test, depending on the expected frequencies in each category; Fisher's exact test was specifically chosen when sample sizes in any category were small (less than 5). Tables were created using the gtsummary package in RStudio [13]. A significance level of p < 0.05 was used for all comparisons.

Data integrity and ethical considerations

This study adhered to the ethical guidelines outlined in the Declaration of Helsinki. Written informed consent was obtained from all participants prior to their inclusion in the study. For the use of patient medical records, participants were informed of the purpose of data collection, the potential risks and benefits, and their right to withdraw from the study at any time without repercussions. To ensure confidentiality and privacy, all patient data were anonymized prior to analysis, and any personally identifiable information (PII) was removed. Data were stored in a secured, password-protected database accessible only to authorized research personnel. No information revealing the identity of the participants was collected or reported. The study was approved by the Institutional Review Board (IRB) of King Saud University Medical City in Riyadh, Saudi Arabia, on July 17, 2023 (reference number: 23/0537).

## Results

Demographic characteristics and comorbidities of patients

The study included 144 known heart failure patients presenting with acute decompensated heart failure with a median age of 66 (interquartile range (IQR): 59.8-77.3) years. The majority were male (68.8%). Among the patients, systemic hypertension was the most common comorbidity (75.7%), followed by diabetes mellitus (63.9%). Other notable comorbidities included asthma (11.1%), chronic kidney disease (18.1%), and stroke or peripheral vascular disease (11.1%). Pulmonary comorbidities such as COPD and pneumonia were less common, each affecting less than 5% of the patients.

In general, 83 (57.6%) patients had CAD. Patients with CAD were younger than those without CAD (median: 66 years (IQR: 63-77.5) versus 67 years (IQR: 42-76), p = 0.026). Males constituted a larger proportion of the CAD group compared to the non-CAD group (75.9% versus 59%, p = 0.031). Diabetes mellitus was significantly more prevalent among patients with CAD (74.7% versus 49.2%, p = 0.002)​​. Other comorbidities did not differ significantly between patients with and without CAD (Table 1).

**Table 1:** Demographic characteristics and comorbidities of patients Median (IQR), number (%) Wilcoxon rank sum test, Pearson's Chi-squared test, Fisher's exact test IQR: interquartile range, CAD: coronary artery disease, COPD: chronic obstructive pulmonary disease, DVT: deep vein thrombosis

Characteristic	Missing	Overall (N = 144)	CAD		p-value
			No (n = 61)	Yes (n = 83)	
Age	0 (0%)	66 (59.8-77.3)	67 (42-76)	66 (63-77.5)	0.026
Gender	0 (0%)				0.031
Male		99 (68.8%)	36 (59%)	63 (75.9%)	
Female		45 (31.3%)	25 (41%)	20 (24.1%)	
Comorbidities					
Systemic hypertension	0 (0%)	109 (75.7%)	43 (70.5%)	66 (79.5%)	0.212
Diabetes mellitus	0 (0%)	92 (63.9%)	30 (49.2%)	62 (74.7%)	0.002
COPD	0 (0%)	1 (0.7%)	1 (1.6%)	0 (0%)	0.424
Asthma	0 (0%)	16 (11.1%)	9 (14.8%)	7 (8.4%)	0.233
Pulmonary embolism	0 (0%)	0 (0%)	0 (0%)	0 (0%)	>0.999
Pneumonia	0 (0%)	4 (2.8%)	2 (3.3%)	2 (2.4%)	>0.999
Other pulmonary disease	0 (0%)	1 (0.7%)	1 (1.6%)	0 (0%)	0.424
Depressive disorder	0 (0%)	2 (1.4%)	2 (3.3%)	0 (0%)	0.178
Stroke or peripheral vascular disease	0 (0%)	16 (11.1%)	4 (6.6%)	12 (14.5%)	0.136
Chronic kidney disease	0 (0%)	26 (18.1%)	7 (11.5%)	19 (22.9%)	0.078
DVT	0 (0%)	2 (1.4%)	1 (1.6%)	1 (1.2%)	>0.999

Clinical signs, symptoms, and laboratory parameters

Chest pain was significantly more common in patients with CAD (38.6% versus 21.3%, p = 0.027). Weight loss was observed only in patients without CAD (8.2% versus 0%, p = 0.012), and lower extremity edema was significantly more frequent in patients without CAD (55.7% versus 37.3%, p = 0.028). Regarding the vital status, patients with CAD had significantly lower heart rates (median: 84 (IQR: 70.5-94) versus 89 (IQR: 77-103), p = 0.031). Additionally, ejection fraction was notably lower in the CAD group, with a greater proportion having an ejection fraction of 40% or less (89.2% versus 62.3%, p < 0.001). Glomerular filtration rate (GFR) was significantly lower in the CAD group (median: 48.3 (IQR: 32-72) versus 66.7 (IQR: 48.2-85.5), p = 0.004). Elevated troponin I levels were more common among CAD patients (25.6% versus 10.3%, p = 0.024) (Table 2)​.

**Table 2:** Clinical signs and symptoms and laboratory parameters among patients Median (IQR), number (%) Pearson's Chi-squared test, Fisher's exact test, Wilcoxon rank sum test IQR: interquartile range, CAD: coronary artery disease, PND: paroxysmal nocturnal dyspnea, JVP: jugular venous pressure, BP: blood pressure, GFR: glomerular filtration rate, BNP: brain natriuretic peptide

Characteristic	Missing	Overall (N = 144)	CAD		p-value
			No (n = 61)	Yes (n = 83)	
Symptoms					
Dyspnea - slight hill	0 (0%)	34 (23.6%)	15 (24.6%)	19 (22.9%)	0.813
Dyspnea - level ground	0 (0%)	32 (22.2%)	15 (24.6%)	17 (20.5%)	0.558
Dyspnea - at rest	0 (0%)	48 (33.3%)	20 (32.8%)	28 (33.7%)	0.905
Dyspnea - PND	0 (0%)	45 (31.3%)	21 (34.4%)	24 (28.9%)	0.481
Chest pain	0 (0%)	45 (31.3%)	13 (21.3%)	32 (38.6%)	0.027
Weight gain	0 (0%)	0 (0%)	0 (0%)	0 (0%)	>0.999
Weight loss	0 (0%)	5 (3.5%)	5 (8.2%)	0 (0%)	0.012
Nocturia	0 (0%)	0 (0%)	0 (0%)	0 (0%)	>0.999
Nausea	0 (0%)	11 (7.6%)	3 (4.9%)	8 (9.6%)	0.356
Coughing	0 (0%)	42 (29.2%)	21 (34.4%)	21 (25.3%)	0.234
Fever	0 (0%)	9 (6.3%)	4 (6.6%)	5 (6%)	>0.999
Expectoration	0 (0%)	24 (16.7%)	14 (23%)	10 (12%)	0.083
Clinical signs					
Tachypnea >20	8 (5.6%)	98 (72.1%)	43 (71.7%)	55 (72.4%)	0.928
Increased JVP	0 (0%)	56 (38.9%)	27 (44.3%)	29 (34.9%)	0.257
Hepatojugular reflux	0 (0%)	0 (0%)	0 (0%)	0 (0%)	>0.999
Rales	0 (0%)	70 (48.6%)	30 (49.2%)	40 (48.2%)	0.907
Wheezing	0 (0%)	8 (5.6%)	3 (4.9%)	5 (6%)	>0.999
Hyperresonant percussion	0 (0%)	0 (0%)	0 (0%)	0 (0%)	>0.999
Dullness	0 (0%)	3 (2.1%)	3 (4.9%)	0 (0%)	0.074
Lower extremity edema	0 (0%)	65 (45.1%)	34 (55.7%)	31 (37.3%)	0.028
Cyanosis	0 (0%)	1 (0.7%)	0 (0%)	1 (1.2%)	>0.999
Vital status					
Systolic BP	0 (0%)	123.5 (111-137.3)	124 (111-139)	122 (111.5-135)	0.944
Diastolic BP	0 (0%)	71 (62.8-84)	74 (64-90)	70 (62.5-80)	0.314
Heart rate	0 (0%)	87 (75-102.3)	89 (77-103)	84 (70.5-94)	0.031
Temperature	1 (0.7%)	36.7 (36.6-36.9)	36.8 (36.6-36.9)	36.7 (36.5-36.9)	0.498
Laboratory findings					
Ejection fraction	0 (0%)				<0.001
50% or more		21 (14.6%)	16 (26.2%)	5 (6%)	
41%-49%		11 (7.6%)	7 (11.5%)	4 (4.8%)	
40% or less		112 (77.8%)	38 (62.3%)	74 (89.2%)	
GFR (≥90 mL/minute/1.73 m^2^)	0 (0%)	55.1 (39.7-78.5)	66.7 (48.2-85.5)	48.3 (32-72)	0.004
Troponin I (mcg/L)	4 (2.8%)				0.024
Normal		113 (80.7%)	52 (89.7%)	61 (74.4%)	
High		27 (19.3%)	6 (10.3%)	21 (25.6%)	
BNP (0-153.5 pg/mL)	2 (1.4%)				0.886
Normal		70 (49.3%)	30 (50%)	40 (48.8%)	
High		72 (50.7%)	30 (50%)	42 (51.2%)	
Hemoglobin level	0 (0%)				0.287
Low		92 (63.9%)	36 (59%)	56 (67.5%)	
Normal		51 (35.4%)	24 (39.3%)	27 (32.5%)	
High		1 (0.7%)	1 (1.6%)	0 (0%)	
Serum albumin level	0 (0%)				0.059
Low		85 (59%)	42 (68.9%)	43 (51.8%)	
Normal		59 (41%)	19 (31.1%)	40 (48.2%)	
High		0 (0%)	0 (0%)	0 (0%)	

Initial outcomes and medications at discharge

A significant difference was observed in ICU admissions, with more CAD patients requiring ICU care (37.3% versus 13.1%, p = 0.001). Upon discharge, CAD patients were more frequently prescribed ACE inhibitors or ARBs (85.5% versus 72.1%, p = 0.047), diuretics (96.4% versus 83.6%, p = 0.008), glyceryl trinitrate (21.7% versus 6.6%, p = 0.013), aspirin (84.3% versus 29.5%, p < 0.001), and anticoagulation (53.0% versus 34.4%, p = 0.027) (Table 3).

**Table 3:** Initial outcomes and medications at discharge Median (IQR), number (%) Wilcoxon rank sum test, Pearson's Chi-squared test, Fisher's exact test IQR: interquartile range, CAD: coronary artery disease, ICU: intensive care unit, CCU: cardiac care unit, MICU: medical intensive care unit, SICU: surgical intensive care unit, ACEI: angiotensin-converting enzyme inhibitor, ARB: angiotensin receptor blocker

Characteristic	Missing	Overall (N = 144)	CAD		p-value
			No (n = 61)	Yes (n = 83)	
Time to treatment (days)	0 (0%)	6 (3-11)	7 (3-13)	5 (3.5-10)	0.300
Length of hospital stay	0 (0%)	6 (3-11)	7 (3-13)	5 (4-9.5)	0.239
Admission to ICU	0 (0%)	39 (27.1%)	8 (13.1%)	31 (37.3%)	0.001
If yes, type of ICU	8 (20.5%)				0.355
CCU		29 (93.5%)	5 (83.3%)	24 (96%)	
MICU		1 (3.2%)	1 (16.7%)	0 (0%)	
SICU		1 (3.2%)	0 (0%)	1 (4%)	
Discharge medication					
ACEI or ARB	0 (0%)	115 (79.9%)	44 (72.1%)	71 (85.5%)	0.047
B-Blocker	0 (0%)	127 (88.2%)	53 (86.9%)	74 (89.2%)	0.676
Diuretic	0 (0%)	131 (91%)	51 (83.6%)	80 (96.4%)	0.008
Glyceryl trinitrate	0 (0%)	22 (15.3%)	4 (6.6%)	18 (21.7%)	0.013
Aspirin	0 (0%)	88 (61.1%)	18 (29.5%)	70 (84.3%)	<0.001
Anticoagulation	0 (0%)	65 (45.1%)	21 (34.4%)	44 (53%)	0.027

Mortality outcomes

At 180 days, 3.6% of patients with CAD had died compared to 0% without CAD (p = 0.262). At 360 days, mortality was 1.6% in the CAD group and 0% in the non-CAD group (p = 0.424), although these differences were not statistically significant​​ (Table 4).

**Table 4:** Mortality outcomes among patients Number (%) Fisher's exact test CAD: coronary artery disease

Characteristic	Missing	Overall (N = 144)	CAD		p-value
			No (n = 61^1^)	Yes (n = 83^1^)	
Death at 180 days	0 (0%)	3 (2.1%)	0 (0%)	3 (3.6%)	0.262
Death at 360 days	0 (0%)	1 (0.7%)	1 (1.6%)	0 (0%)	0.424

## Discussion

Our study aimed to assess how coronary artery disease (CAD) affects the short-term outcomes of patients with acute decompensated heart failure (ADHF) in the emergency department. Additionally, the study sought to identify key differences in demographics, clinical parameters, and outcomes, including ICU admissions and medications, between patients with and without CAD. By comparing these two groups, we found significant differences in comorbidities, clinical signs and symptoms, laboratory tests such as GFR, and ICU admissions, underscoring the need for tailored treatment approaches as the prevalence of CAD and ADHF continues to rise.

Our study found systemic hypertension as the most common comorbidity (79.5%), followed by diabetes mellitus (74.7%), with diabetes being more prevalent in CAD (p = 0.002). Comparatively, Purek et al. (2006) reported systemic hypertension in 64% and diabetes mellitus in 31% of their heart failure patients [7]. This discrepancy might be due to differing population demographics and the rapid increase in diabetes and hypertension prevalence. This is supported by multiple pooled analyses as both studies attributed the rise to better detection and rising prevalence of risk factors such as obesity [14,15]. Also, our findings align with a similar study done in Saudi Arabia in 2013 that reported the prevalence of hypertension and diabetes to be 64% and 71%, respectively [16]. Additionally, unexamined social factors, such as socioeconomic status, healthcare access, and lifestyle, could influence these outcomes, as these factors can significantly impact the prevalence of comorbidities. Our findings of a higher prevalence of diabetes mellitus underscore the significant burden of this comorbidity in our population. A study done in 2001 on normotensive, asymptomatic, well-controlled diabetes type 2 to assess the prevalence of diastolic dysfunction found the diagnosis of diastolic dysfunction in 47% [17], which further implies the burden. Multiple antidiabetic medications were found to play a huge role in the prognosis of HF, especially sodium-glucose cotransporter-2 (SGLT2) inhibitors. It was found that SGLT2 inhibitors decrease the odds of all-cause mortality, cardiovascular mortality, heart failure events, and readmission rates within the first 1-9 months of hospitalization [18].

One of the interesting findings of our study is that patients with ADHF and CAD were younger than those without CAD (median age: 66 years versus 67 years, p = 0.026). This goes in contrast with the existing literature, which typically shows no significant difference in age or even older age in CAD patients with heart failure. For instance, a study done in 2013 reported that acute heart failure patients with acute coronary syndrome (ACS) were generally older [19]. Additionally, Purek et al. (2006) found that age did not significantly differ between heart failure patients with and without CAD [7]. However, data from the Kyoto Congestive Heart Failure (KCHF) Registry reported in 2022 to compare acute heart failure patients who were hospitalized and undergone coronary angiography versus those who were not reported that people who underwent coronary angiography were generally younger (72.3 ± 12.2 versus 80.1 ± 11.5, p = 0.001) [20]. Our findings may be attributed to several factors. One important factor is the possibility that the earlier onset of CAD symptoms and complications in younger patients leads to an earlier diagnosis of both CAD and heart failure. This is possibly linked to lifestyle choices and genetic predispositions. Lifestyle factors, such as higher rates of smoking, poor dietary habits, and limited physical activity, are prevalent in our region and contribute to the earlier development of CAD [3]. Furthermore, genetic predispositions, such as a higher prevalence of metabolic syndrome as noted in the aforementioned study [14], may accelerate atherosclerosis in younger individuals. The high prevalence of diabetes and obesity in our population also exacerbates the risk of early CAD development. These metabolic and lifestyle factors, combined with earlier detection and diagnosis, may account for the younger age distribution of CAD in our ADHF patients. A recent study was done in 2023 to compare the long-term outcomes following early CAD testing in new-onset heart failure and concluded that prompt evaluation for CAD after new HF resulted in a modest reduction of all-cause mortality, which was mainly due to increased subsequent statin use, revascularization, and other cardiovascular interventions such as stroke prophylaxis for atrial fibrillation [21]. The importance of early testing is also highlighted by another study done in 2020, which concluded that testing for CAD was low among older patients diagnosed with HF and that in patients with left ventricular ejection fraction of less than 40%, testing was higher [22]. This age difference could be significant for clinical practice, highlighting the need for prompt interventions and guideline-directed medical therapy of CAD to prevent heart failure in younger populations.

Our study found that males made up a larger proportion of the CAD group compared to the non-CAD group (75.9% versus 59%, p = 0.031). This is consistent with previous research showing that males are generally more prone to developing coronary artery disease (CAD) than females [23]. Hormonal differences, such as the protective effect of estrogen in females, especially before menopause, help explain this [24]. Males are also more likely to have lifestyle risk factors such as smoking, which contribute to a greater risk of CAD [25]. These gender differences highlight the need for considering gender-specific factors when managing CAD and heart failure.

The presence of chest pain and weight loss can influence both diagnosis and treatment decisions in patients with acute decompensated heart failure (ADHF). Chest pain, significantly more common in CAD patients (38.6% versus 21.3%, p = 0.027), typically suggests myocardial ischemia or infarction. This leads to a focus on ruling out acute coronary syndrome (ACS) and can prompt aggressive interventions such as revascularization or adjustments in ischemia-directed therapies. On the other hand, weight loss, seen only in non-CAD patients (8.2% versus 0%, p = 0.012), may indicate heart failure-related cachexia or malnutrition [26]. This shifts the clinical approach toward optimizing nutritional support and careful fluid management, addressing non-ischemic causes and comorbidities that may contribute to the patient's condition. Also, our study found that edema was significantly more common in patients without CAD (55.7% versus 37.3%, p = 0.028). This result when compared with prior literature was found to be statistically insignificant [19,27].

We also observed that ejection fraction (EF) was notably lower in the CAD group, with a greater proportion having an EF of 40% or less (89.2% versus 62.3%, p < 0.001). This aligns with findings from Drexler et al. (2012), who reported lower EF in ischemic acute heart failure patients. The lower EF in CAD patients highlights the severe impact of ischemic damage on cardiac function [28]. Moreover, in the Efficacy of Vasopressin Antagonist in Heart Failure Outcome Study With Tolvaptan (EVEREST) trial, Mentz et al. (2013) found that while all-cause mortality was the same, documented CAD in HF with reduced ejection fraction was linked with higher comorbidity burden, HF hospitalization, and lower use of HF medications [19]. Also, in a study that reported the response to treatments in recent-onset heart failure with reduced ejection fraction (HFrEF) in ischemic versus non-ischemic etiologies, those with non-ischemic etiologies responded better to guideline-directed therapy [29].

Our study showed that the glomerular filtration rate (GFR) was significantly lower in the CAD group (median: 48.3 versus 66.7, p = 0.004), indicating worse renal function. As it has been established before, as the GFR declines, the incidence of CAD increases [30,31]. This is supported by the fact that, particularly at an estimated GFR < 15 mL/minute/1.73 m^2^, there is an inverse and independent correlation between renal function and cardiovascular morbidity and mortality [32,33]. Troponin levels were also higher in CAD patients in our study (25.6% versus 10.3%, p = 0.024). Similar findings were reported in other studies, emphasizing that elevated troponin levels are a marker of myocardial injury and are more prevalent in ischemic heart failure [28,34]. Braga et al. (2013) mentioned that those with higher levels of cardiac troponins were associated with a higher risk of death and hospitalizations [35]. On the other hand, an RCT was done on AHF patients presenting with elevated troponin levels not due to acute coronary syndrome. Their primary outcome was 30-day mortality, and they found that elevated troponins were not associated with the primary outcome [36]. The difference in mortality may stem from ACS patients having more direct ischemic injury, as noted by Braga et al. (2013) [35], whereas elevated troponins in non-ACS ADHF likely reflect myocardial stress rather than acute ischemia. The shorter follow-up in the RCT may also have missed long-term risks associated with elevated troponins. The aforementioned results along with ours highlight the need for in-depth investigations in patients presenting with ADHF.

Our study found that ICU admissions were significantly higher in CAD patients (37.3% versus 13.1%, p = 0.001). This finding is consistent with the study done in Saudi Arabia by Albackr et al. (2013), which showed higher ICU admission rates for patients with CAD and heart failure, reflecting the severity and complexity of managing these patients [16]. Similarly, Drexler et al. (2012) reported that heart failure patients with ischemic heart disease often require more intensive care, highlighting the need and impact of specialized management strategies [28]. This has important clinical implications, as higher ICU admissions in CAD patients may strain hospital resources, requiring more intensive care staff and equipment. Early identification and aggressive management of high-risk CAD patients can help reduce ICU stays by stabilizing them earlier in their hospital course. This finding reflects the increased severity and complexity of CAD in the context of acute decompensated heart failure (ADHF). CAD patients often experience more severe myocardial ischemia and having comorbidities such as reduced ejection fraction and impaired renal function as indicated by lower GFR in our study. These factors contribute to the need for more intensive monitoring and management, leading to higher ICU admissions.

Regarding medications at discharge, our study observed that CAD patients were more frequently prescribed ACE inhibitors or ARBs (85.5% versus 72.1%, p = 0.047), diuretics (96.4% versus 83.6%, p = 0.008), glyceryl trinitrate (21.7% versus 6.6%, p = 0.013), aspirin (84.3% versus 29.5%, p < 0.001), and anticoagulation (53% versus 34.4%, p = 0.027). This aligns with current international treatment guidelines. These findings are in accordance with a previous study done in Saudi Arabia in 2016 that showed similar results and discharge medications, except for diuretics, which were given more in non-CAD patients in contrast to our study [34]. This is also supported by a study done in 2014 [37]. Diuretics such as loop diuretics and aldosterone antagonists are often indicated to decrease the load of the overloaded circulation, and others such as thiazides are often indicated for hypertension [38]. This may be implicated as CAD patients are often associated with more morbidity in terms of ICU admissions and decreased levels of GFRs and, hence, more usage of diuretics in these patients.

Our results showed that CAD patients had a higher risk of mortality at 180- and 360-day marks; however, it was deemed statistically insignificant. This finding goes in line with the existing literature, which has often shown that CAD is associated with worse short-term outcomes in heart failure patients. One possible reason for the lack of statistically significant mortality differences could be the relatively short follow-up period, which may not have been sufficient to detect the longer-term effects of CAD on mortality. Additionally, intensive management of both CAD and non-CAD patients during hospitalization may have mitigated mortality differences in the short term. Both groups received guideline-directed medical therapy, which likely helped improve outcomes for all patients, regardless of CAD status. For example, a study done in 2014 found that patients with acute heart failure precipitated by acute coronary syndromes (ACS-AHF) had significantly higher 30-day mortality compared to those without ACS, although long-term survival rates were similar between the two groups​ [37]. This finding is also supported by many other studies [7,20,34,39]. This implies that future studies should focus more on interventions that can alter the overall short-term mortality. Many studies tried to assess the early initiation of increased intensity guideline-directed medical therapy or early angiography and their effects on outcomes. All showed improved survival and decreased mortality [40,41].

Strengths and limitations

Our study has several strengths, including its prospective cohort design, which allowed for systematic data collection and minimized recall bias. This design ensures a more reliable capture of patient data over time, reducing potential inaccuracies that retrospective studies might face. We gathered comprehensive data, including a broad range of clinical parameters such as demographics, signs and symptoms, and laboratory parameters, providing a robust dataset for analysis.

However, our study has limitations. Being a single-center study may limit the generalizability of our findings. Differences in patient demographics, such as ethnicity or socioeconomic factors, and variations in clinical practices between regions and hospitals may affect the broader applicability of our results. Due to the short follow-up period, we could not assess long-term mortality outcomes. This limitation likely led to an underestimation of the true impact of CAD on long-term survival in ADHF patients. Without sufficient follow-up time, we may have missed later mortality events that could have provided a clearer distinction between CAD and non-CAD outcomes. Moreover, this limitation prevented us from performing a Kaplan-Meier survival analysis, which would have offered a more comprehensive comparison of survival rates between the groups. As a result, the interpretation of our survival data should be approached with caution, particularly when considering CAD's potential long-term effects. Additionally, our study did not specifically distinguish between acute coronary syndrome (ACS) and other types of CAD in our patient group. Future research should consider making this distinction, as ACS might have a different impact on short- and long-term outcomes compared to stable CAD. Understanding these differences could provide clearer insights into how each condition influences heart failure progression. Researchers should explore how differentiating between different types of ACS and stable CAD affects the management and prognosis of patients with acute decompensated heart failure (ADHF). These limitations might affect the robustness of our conclusions. Survival outcomes, in particular, could lead to an underestimation of the true burden and effect of CAD on the survivability of ADHF patients. Nonetheless, the consistency of our results with existing literature supports their validity and underscores the critical role of CAD management in ADHF patients. Another limitation of our study is that we did not categorize patients by ejection fraction. We recommend researchers explore this to gain deeper insights into prognosis and treatment in ADHF patients with CAD.

Future research should aim to validate our findings in larger, multicenter trials to ensure generalizability. Additionally, longer follow-up periods will be crucial in assessing the long-term impact of CAD on ADHF. Studies should also explore the utility of advanced imaging techniques, such as cardiac MRI, which allows for detailed assessment of myocardial fibrosis, and coronary CT angiography for detecting subclinical atherosclerosis. These imaging modalities can play a key role in the early detection and management of CAD in ADHF patients, offering critical insights that may improve patient outcomes [42]. Research focusing on specific CAD interventions and their long-term benefits will help develop optimal treatment protocols. Furthermore, investigating the impact of comorbid conditions on the prognosis of ADHF patients with CAD can provide further insights into personalized treatment approaches. Rossi et al. (2008) suggest that incorporating advanced imaging and biomarker assessments can aid in better classification and management of ADHF patients, which aligns with our findings [39]. This was also highlighted by another study that signified the underutilization of ischemic CAD testing in new-onset HF [43].

## Conclusions

Our study shows that CAD significantly influences ADHF outcomes, with younger age distribution, male predominance, and higher rates of hypertension and diabetes among CAD patients. Early and targeted management of CAD is essential to improve clinical outcomes in this population. Future research should focus on the long-term effects of CAD on mortality in ADHF and explore the specific impacts of different types of ACS on both short- and long-term outcomes in CAD patients.
